# Case report: A non-obstructive azoospermia patient with heat shock factor-2 mutation

**DOI:** 10.1097/MD.0000000000021107

**Published:** 2020-07-31

**Authors:** Haiyue Zhao, Hongguo Zhang, Qi Xi, Leilei Li, Haibo Zhu, Xiaonan Hu, Ruizhi Liu

**Affiliations:** Centre for Reproductive Medicine and Prenatal Diagnosis, First Hospital of Jilin University, Changchun, P.R. China.

**Keywords:** heat shock factor-2, male infertility, mutation, next-generation sequencing, non-obstructive azoospermia

## Abstract

**Rationale:**

Infertility is a common medical condition that affects nearly 15% of the world population. Non-obstructive azoospermia (NOA) is one of the most severe forms of male infertility. Some common structural variants, single nucleotide polymorphisms (SNPs), and genetic factors were reported to be associated with NOA. However, the underlying etiology and genetic mechanism(s) remain largely unclear. This report aimed to describe the associated mutation of the heat shock factor-2 (HSF2) gene in Chinese infertile men with NOA.

**Patient concerns:**

An apparently healthy 27-year-old man with a body mass index (BMI) of 23.31 kg/m^2^ had a 2-year history of primary infertility.

**Diagnoses:**

The semen analysis of the patient showed a sperm concentration of 0/mL in 6.5 mL of semen. The patient was diagnosed with NOA by performing the comprehensive examinations including a detailed medical history, physical examination, chromosome analysis, Y-chromosome microdeletions, semen analysis, and hormone profiles.

**Interventions:**

The couple received artificial insemination by donor (AID) and a healthy girl was born after the embryo transfer.

**Outcomes:**

We found a novel deletion-insertion variation c.326_326delinsGGAAGGTGAGCTATTGT in the exon 3 of the HSF2 gene by performing next-generation sequencing on him who was diagnosed NOA. We performed Sanger sequencing on this patient and confirmed the heterozygous missing insertion mutation in the patient. This is a novel mutation. The variant was heterozygous and categorized as pathogenic.

**Lessons:**

A novel deletion-insertion variation c.326_326delinsGGAAGGTGAGCTATTGT in the exon 3 of HSF2 gene HSF2 is predicted to be pathogenic and associated with the occurrence of NOA.

## 1 Introduction

Infertility affects about 15% of couples who wish to have children and half of these cases are associated with male factors. Non-obstructive azoospermia (NOA) is one of the most severe forms of male infertility because of impaired spermatogenesis with the absence of spermatozoa in the ejaculate.^[1]^ The etiology of NOA is either intrinsic testicular impairment or inadequate gonadotropin production. Chromosomal or genetic abnormalities should be evaluated because the prevalence of chromosome abnormalities is higher in infertile men, this figure being inversely related to the sperm count. Based on the largest published series, it could be estimated that the overall incidence of a chromosomal factor in infertile men ranges between 2% and 8%, with a mean value of 5%. This value increases to about 15% in azoospermic men.^[2]^ This case report mainly describes the clinical manifestations and treatment outcomes of NOA patients with a new heat shock factor-2 (HSF2) mutations on chromosome 6 and explores the pathogenesis of NOA by combining with other studies on the HSF2 gene.

Heat shock factors (HSFs), as regulators of heat shock proteins (HSPs) expression, are well known for their cytoprotective functions during cellular stress. There are 4 subtypes of HSFs found in eukaryotes: heat shock factor-1 (HSF1), heat shock factor-2 (HSF2), heat shock factor-3 (HSF3), and heat shock factor-4 (HSF4).^[3]^ All heat shock factor (HSF) family members are expressed during mammalian spermatogenesis, mainly in spermatocytes and round spermatids which are characterized by extensive chromatin remodeling. Different HSFs could cooperate to maintain proper spermatogenesis. There has been a significant amount of research attempting to elucidate the biological role(s) of HSF1 and HSF2. Some of this research has used mice with mutations in these genes to identify their function. There is good agreement in the literature regarding the importance of HSF1 and HSF2 for male and female fertility.^[4–6]^

## 2 Methods

The patient was diagnosed as having NOA by performing the comprehensive examinations. He was subjected to sequencing analysis of the spermatogenesis associated genes. Genomic DNA was isolated from blood lymphocyte samples and subjected to exome capture using the in the house Targeted genes Panel (Peking Medriv Academy of Genetics and Reproduction, Peking) followed by next-generation sequencing on the Illumina MiSeq sequencing platform. The mutation is validated by sanger sequencing. Primers for the detected mutation c.326_326delinsGGAAGGTGAGCTATTGT in HSF2 were 5′-TACTGATGAAGCCATTT-3′ (forward) and 5′-CATATTCTAGCAGGAACT-3′ (reverse). The original amino acid sequence of HSF2 was searched from the human reference genome (GRCh37/hg19), and located the mutation site according to the second-generation sequencing results. The amino acid coding sequence after mutation of HSF2 was predicted by the EMBOSS-Transeq online tool.

## 3 Case description

A 27-year-old man came to our hospital for treatment because of infertility. By performing the comprehensive examinations including a detailed medical history, physical examination, chromosome analysis, Y-chromosome microdeletions, semen analysis, and hormone profiles, the patient was diagnosed as having NOA. He was an otherwise healthy worker with a body mass index (BMI) of 23.31 kg/m^2^. He had normal virilization and normal potency. His physical examination including the external genitalia was essentially normal excepting bilateral testicular dysplasia. Evaluation of the partner indicated his wife had a chocolate cyst of the ovary. He does not smoke, does not drink alcohol, and has no history of drug allergies. Further evaluation revealed a low seminal fluid volume and no sperm, estradiol, follicle-stimulating hormone (FSH), luteinizing hormone (LH), and prolactin (PRL) levels were normal and reduced testosterone levels. The azoospermia factor (AZF) microdeletion test results and karyotype are normal. A contrast-enhanced Computed Tomography scan of chest, abdomen, and pelvis and magnetic resonance imaging (MRI) scan of the pituitary fossa were normal too. The couple choose to accept donated spermatozoa. Subsequently, the pregnancy resulted in the live birth of a girl at 37 weeks. The study was approved by the Ethics Committee of the First Hospital of Jilin University. Each participant provided written informed consent before diagnosis. The patient has provided informed consent for publication of the case.

**Figure 1:**
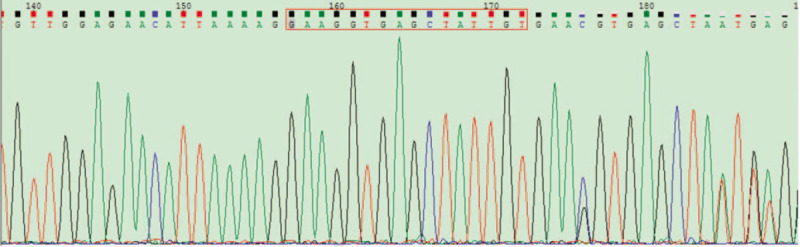
The HSF2 c.326_326delinsGGAAGGTGAGCTATTGT mutation in patient was confirmed by Sanger sequencing. HSF2 = heat shock factor-2.

We found a novel deletion-insertion variation c.326_326delinsGGAAGGTGAGCTATTGT in the exon 3 of the HSF2 gene by performing next-generation sequencing on him who was diagnosed NOA. We performed Sanger sequencing on this patient and confirmed the heterozygous missing insertion mutation in the patient (Fig. 1). This is a novel variation that has not been reported before. The National Center for Biotechnology Information (NCBI) database was used to predict the transcription of the gene after mutation, and it was found that the amino acid sequence encoded by HSF2 was changed from normal 518 to 124, and transcription was terminated prematurely. And among the 124 amino acids transcribed, a mutation occurred from the 113th amino acid, 113 amino acids were mutated from Ser to Tyr. The 113th to 124th amino acid sequence after mutation is YCEGFIFKTRRK, after which the amino acids are lost. We predicted the variant to be pathogenic by mutation site. Scrotal color Doppler ultrasonography of this patient revealed that his testicular had a homogeneous echotexture and wide hypoechogenicity and bilateral testicular dysplasia with the volume was 6.9 mL left and 4.3 mL right. No solid or cystic lesions were observed. The clinical and hormone data of this patient were summarized in Table 1. Finally, the couple received artificial insemination by donor (AID) and a healthy girl was born after the embryo transfer.

**Table 1:**
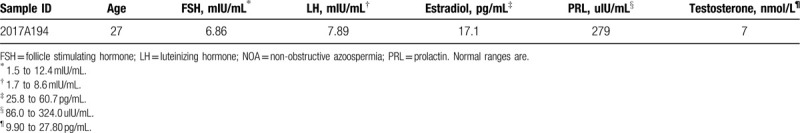
Clinical and hormone profile of patient with NOA with novel HSF2 deletion-insertion mutation.

## 4 Discussion

Many factors are known to cause male infertility. NOA as one of the most severe forms of male infertility has been extensively researched. Chromosomal abnormalities are perhaps best known to interfere with spermatogenesis. Known reported in the pathogenesis of NOA are No. 5, No. 6, No. 10, and Y chromosome.^[7–10]^ Genes that have been reported on the Y chromosome included AZF, zinc finger protein, Y linked (ZFY), amelogenin, Y-linked (AMELY), transducin Beta-like 1Y (TBL1Y), testis-specific protein Y-linked (TSPY), etc.^[10]^ The AZF region on the Y chromosome is one of the most intensively studied regions in male infertility.^[11,12]^ The deletion of the AZFc region of the Y chromosome is the most frequent molecularly defined cause of spermatogenic failure. It was known that the translocations of chromosomes 5, 6, and 10 were also associated with NOA. The karyotype of NOA patients is 46,XY,t(6;7) (q15;p15), and the breakpoints at 10p12 and 10q26.3 were considered to be associated with pregestational infertility.^[7,9]^ These genetic defects impede the development of the male gonads or urogenital tract during development, cause arrest of germ cell production and/or maturation or produce non-functional spermatozoa. Our case report also found that NOA is related to the gene on chromosome 6. Unlike previous reports, we found a deletion-insertion variation in the HSF2 gene. Located in exon 3, is a novel variation. The occurrence of this mutation results in the early termination of the wild-type encoded amino acid sequence, which is pathogenic.

HSFs function as transcriptional regulators of HSPs gene expression in eukaryotic cells. HSF2, belonging to the family of HSFs, HSFs regulate the dynamic expression of different HSPs that are responsible for subsequent downstream effects, including stress-related cytoprotective functions, folding, and assembling of nascent polypeptides, and intracellular transport of proteins.^[13,14]^ Researches shown that both the expression and functional properties of HSF2 are regulated in mouse testis cells.^[13,15]^ At least 2 splice forms, HSF2a and HSF2b, were identified for HSF2 and the HSF2a gene was expressed predominantly in the mouse testis, the testis expressing primarily the larger HSF2a protein isoform and the heart and brain expressing predominantly the smaller HSF2b protein isoform. Studies have shown that the level of HSF2a mRNA isoforms in the testes increases 7.6 times in a short period after birth.^[13]^ Bilateral testicular dysplasia may be associated with disruption of HSF2 gene function. According to researches define a physiological role for HSF2 in the regulation of mouse Y chromosome long arm (MSYq) resident genes and the quality of sperm. The male-specific region of the MSYq, which harbors multiple copies of a few genes, the deletion of it will lead to sperm head defects and impaired fertility. The disruption of HSF2 caused a similar phenotype as the 2/3 deletion of MSYq. Our findings define a physiological role for HSF2 in the regulation of MSYq resident genes and the quality of sperm.^[16]^ The analyses of the HSF2 knockout phenotype, that is, sperm head morphology defect together with altered chromatin packing protein levels and increased DNA damage, indicate that HSF2 is critical for sperm differentiation and correct packing of the chromatin in the male germ cells.^[16]^ The above results indicate that HSF2 is associated with the occurrence of sperm and is consistent with the clinical manifestations of the patients we reported.

HSPs are transcriptionally regulated by HSFs, maintain protein homeostasis and promote cell survival. All HSF family members are expressed during mammalian spermatogenesis, mainly in spermatocytes and round spermatids.^[6]^ Different HSFs could cooperate to maintain proper spermatogenesis. The cooperation of HSF1 and HSF2 is especially well established. These 2 factors can directly bind each other and form heterotrimers regulating the basal level of transcription of target genes.^[17,18]^ But both structural and mechanistic insights into HSF1–HSF2 heterotrimerization are very limited.^[19–21]^ Their double knockout results in meiosis arrest, spermatocyte apoptosis, and male infertility. Because both factors are involved in the repackaging of the DNA during spermatid differentiation. HSF2-knockout results in mice exhibit defects in spermatogenesis, and men exhibit reduced fertility a few months after birth and in the background of HSF1 deficiency, all men are infertile due to complete disruption in spermatogenesis.^[22]^ The above findings are consistent with our predictions that the occurrence of HSF2 deletion-insertion variation not only affects its function but also affects the formation of HSF1 complex. Both of these reasons may affect spermatogenesis and even lead to apoptosis of spermatogonia.

The limitation of this report is that we cannot perform any functional study to demonstrate the pathogenicity of the variant. Although we have not yet made a definite inference about the consequences of the missing insertion mutation c.326_326delinsGGAAGGTGAGCTATTGT in HSF2, what is clear is that the mutation results in the loss of residues after 124th amino acids. Therefore, the novel missing insertion mutation was likely to affect the function of HSPs protein. This speculation is consistent with the results reported in other related literature. In a word, our study revealed a novel missing insertion mutation c.326_326delinsGGAAGGTGAGCTATTGT in HSF2 which expanded the mutation spectrum of HSF2 in Chinese NOA infertile men and advanced our understanding of the genetic susceptibility to NOA.

## Author contributions

**Funding acquisition:** Ruizhi Liu.

**Investigation:** Hongguo Zhang, Qi Xi, Leilei Li.

**Methodology:** Haibo Zhu, Xiaonan Hu.

**Writing – original draft:** Haiyue Zhao.

**Writing – review & editing:** Ruizhi Liu.
